# P-318. HIV Pre-Exposure Prophylaxis Eligibility and Feasibility in the Health Frontiers in Tijuana Network

**DOI:** 10.1093/ofid/ofaf695.537

**Published:** 2026-01-11

**Authors:** Matthew Cappiello, Casey Gaughan, Samvel Gaboyan, Carlos Antonio Garcia Tovar, Miguel Prado, Jennifer Veltman, Jose Luis Burgos

**Affiliations:** Loma Linda University, Temecula, CA; Loma Linda University School of Medicine, San Bernardino, CA; University of California, San Diego, San Diego, California; UC San Diego School of Medicine, La Jolla, California; UC San Diego, Lemon Grove, California; Loma Linda University School of Medicine, San Bernardino, CA; UCSD School of Medicine, UCSD Global Health Program, UCSD Herbert Wertheim School of Public Health and Human Longevity Science, San Diego, California

## Abstract

**Background:**

The Tijuana-California border is a metroplex that integrates multiple risk factors for HIV transmission across borders. However, awareness and uptake of pre-exposure prophylaxis (PrEP) remains low in the region. Health Frontiers in Tijuana (HFiT), a partnership between United States and Tijuana academic training sites for marginalized populations, may serve as an optimal location for PrEP delivery.

**Table 1:**
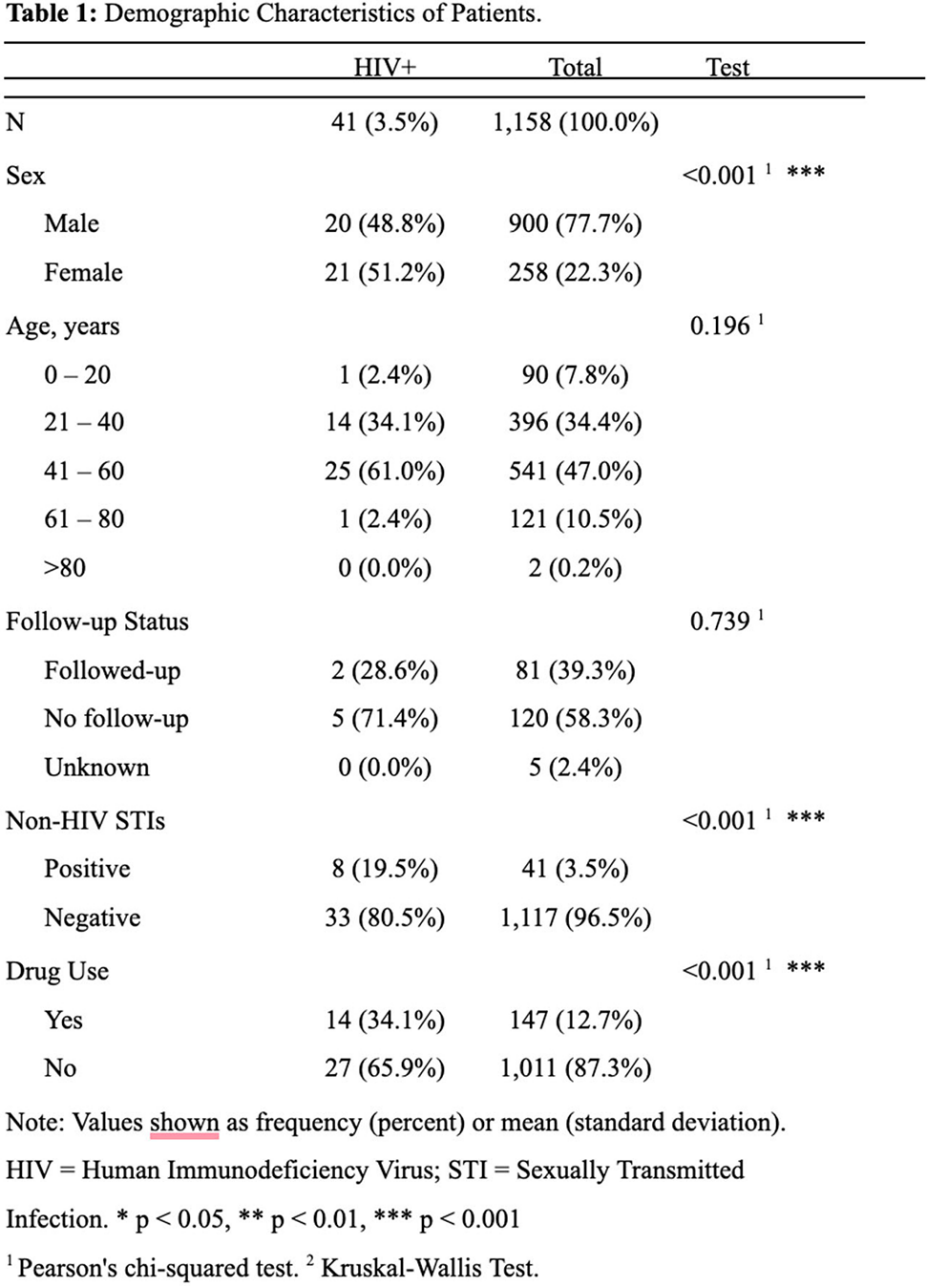
Demographic Characteristics of Patients

**Table 2:**
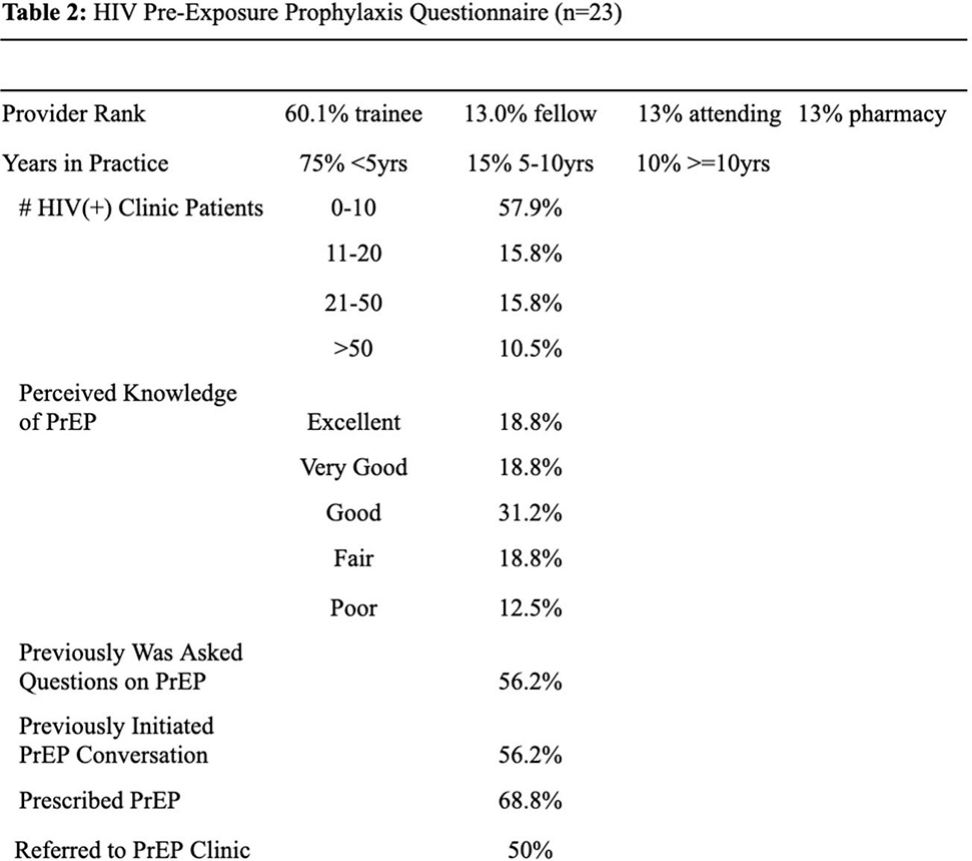
HIV Pre-Exposure Prophylaxis Questionnaire: Demographics

**Methods:**

We assessed risk factors for HIV transmission among electronic medical record patient encounters (n=2390) from January 2022 to December 2024 (Table 1). Anonymized surveys were conducted from January through April 2025 (n = 23) to assess PrEP delivery capacity among HFiT-affiliated physicians and pharmacists (Tables 2 and 3). This survey was derived from prior questionnaires on PrEP awareness in health providers.

**Table 3:**
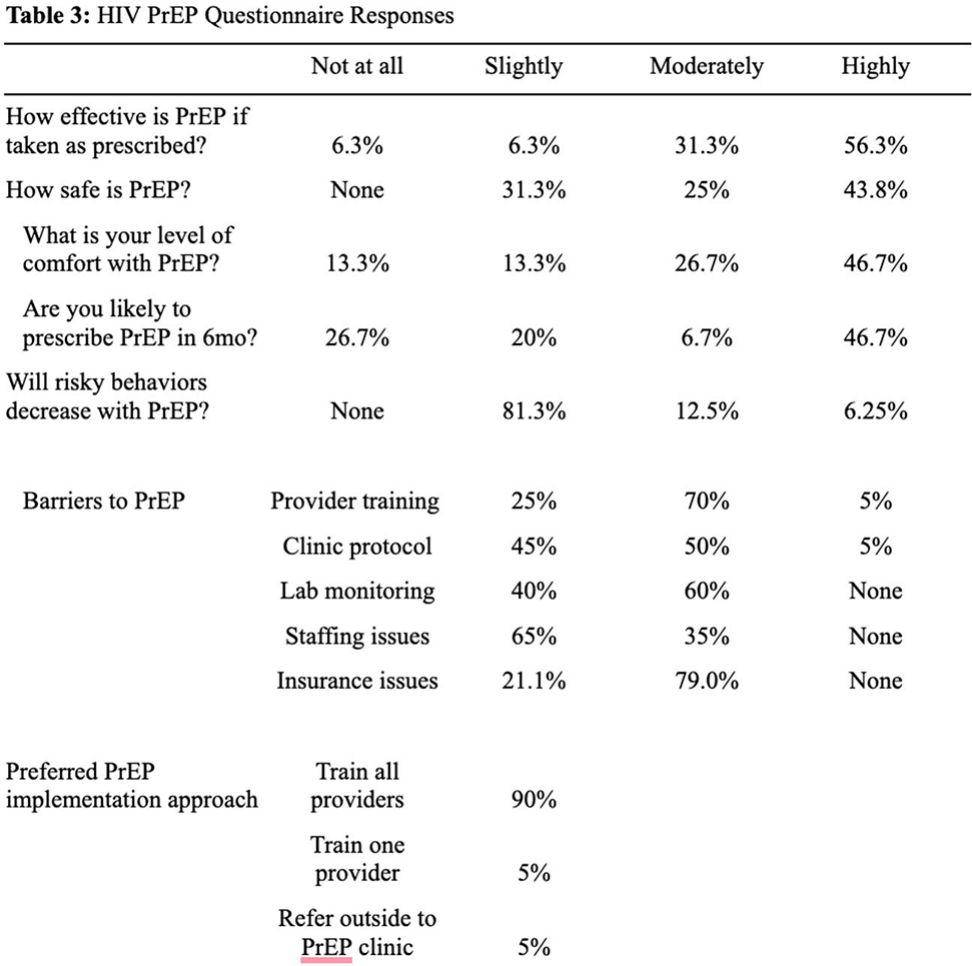
HIV Pre-Exposure Questionnaire: Barriers and Facilitators

**Table 4:**
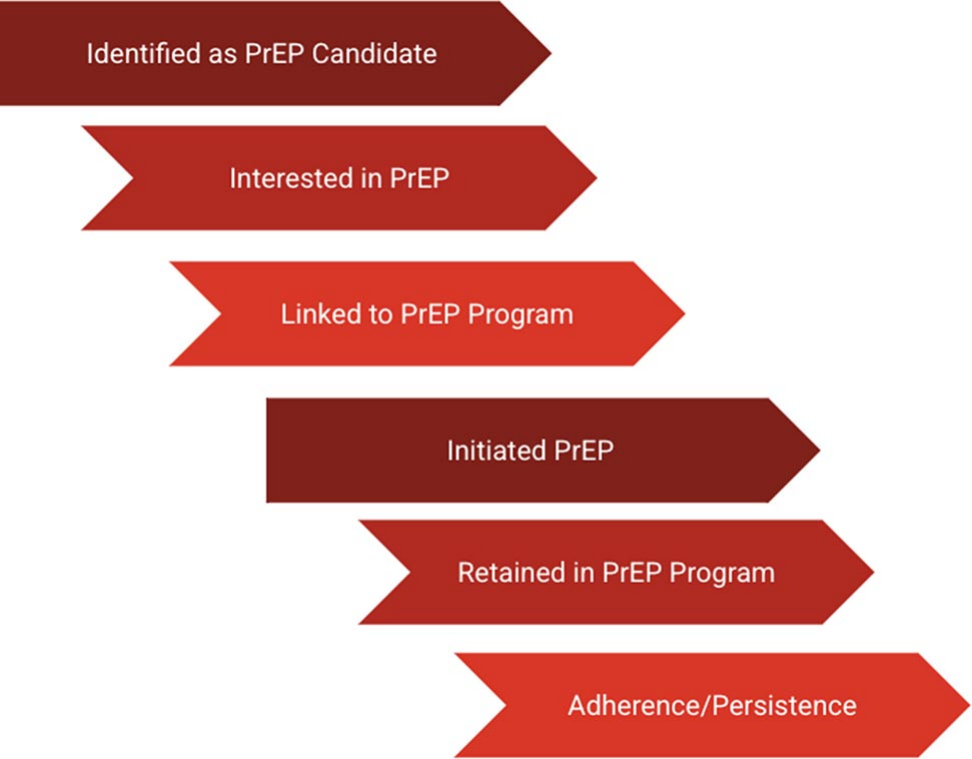
PrEP Care Continuum

**Results:**

HIV prevalence in this population (3.5%) was over 18 times the estimated national HIV prevalence in Mexico, and more than four times above Baja California background rates. Female gender, use of recreational drugs, and presence of comorbid sexually transmitted infections (p< 0.001) were associated with increased risk for HIV positive test. This data suggests a high number of eligible patients for PrEP. A substantial number of survey respondees (87.6%) felt that PrEP was of high utility in HFiT’s patient population. Both physicians (68.8%) and pharmacists (100%) had an adequate understanding of which patients were eligible for PrEP. Education gaps regarding PrEP eligibility were seen in junior providers such as medical students, but less so in senior providers such as residents and attending physicians. Barriers to PrEP adoption appeared feasible to overcome. Provider perception regarding insurance ineligibility (79%) has been mitigated by recent approval of tenofovir disoproxil fumarate-emtricitabine in Mexican public insurance. Concerns about staffing (35%) and lab barriers (60%) can also be mitigated, through provider education materials and identification of discrete sites in the PrEP care continuum (Table 4).

**Conclusion:**

Overall, high eligibility for PrEP exists in HFiT’s population. Future uptake interventions at this site can lessen HIV spread in Tijuana, including development of a PrEP care continuum for the Tijuana metropolitan area.

**Disclosures:**

All Authors: No reported disclosures

